# Study of a Two-Step Centrifugation Protocol for Concentrating Cells and Growth Factors in Bovine Platelet-Rich Plasma

**DOI:** 10.1155/2017/1950401

**Published:** 2017-10-30

**Authors:** Claudia M. Gutiérrez, Catalina López, Carlos E. Giraldo, Jorge U. Carmona

**Affiliations:** Grupo de Investigación Terapia Regenerativa, Departamento de Salud Animal, Universidad de Caldas, Manizales, Colombia

## Abstract

There is a lack of information about the methods used for bovine platelet-rich plasma (PRP)/platelet-rich gel (PRG) procurement, including information on platelet (PLT), white blood cell (WBC) in PRP, and growth factor release from PRG supernatants. The aims of this study were to compare and to correlate the PLT, WBC, transforming growth factor beta-1 (TGF-*β*_1_), and platelet-derived growth factor BB (PDGF-BB) concentrations in bovine whole blood, plasma, and four PRP layers and their respective PRG supernatants: A and B (obtained by a single centrifugation tube method at 720*g*/5 min) and C and D (obtained by a double centrifugation tube method, by using two centrifugation episodes at 720*g*/5 min). PLT and WBC counts were significantly higher in PRP-C, followed by whole blood, PRP-A, PRP-B, and PRP-D. TGF-*β*_1_ concentrations were significantly higher in PRG-B supernatants and its correspondent PRP-B lysate when compared to the other PRG supernatants and plasma. Supernatants from PRG-A, PRG-B, and PRG-D had equivalent TGF-*β*_1_ concentrations. PDGF-BB concentrations were not statistically different between the hemoderivatives. Significant Pearson correlations were noted between PLT counts and WBC counts (0.8) and between PLT counts and PLT distribution width (0.6). Further studies should be performed to assess the potential clinical applications of these PRPs.

## 1. Introduction

Platelet-rich plasma (PRP), a plasma preparation with variable concentrations of platelets (PLTs) and white blood cells (WBCs), is a biomaterial rich in growth factors (GFs), such as transforming growth factor beta-1 (TGF-*β*_1_) and platelet-derived growth factor type BB (PDGF-BB) [1]. Currently, PRP (liquid) compounds are classified into two groups according to the presence or absence of WBCs, that is, leukocyte-concentrated PRP (Lc-PRP) and leukocyte-reduced PRP (Lr-PRP), also known as pure-PRP (P-PRP) [2]. Once these PRPs are mixed with a platelet activating substance (i.e., calcium salt or thrombin) or come into contact with tissue collagen, they change into a gel form, that is, platelet-rich gel (PRG), which gradually releases GF. Notably, the cell composition of the PRP will determine the GF and cytokine profile released from activated PLTs and WBCs [3].

PRP types, either Lc-PRP or Lr-PRP, are currently used (among other uses) for the treatment of several musculoskeletal injuries and as a surgical coadjuvant in both human [4] and veterinary [5] medicine. In general, the rationale behind the use of this biomaterial lies in the fact that the GF and cytokines contained within are gradually released and diminish inflammation, promote neovascularization, increase extracellular matrix protein deposition, and induce cell migration and proliferation [4, 6]. In other words, these substances could enhance tissue regeneration [7].

PRP has been mostly used in horses [6, 8] and dogs [9, 10] for the treatment of tenodesmic lesions and joint diseases. Furthermore, there are studies about the use of several PRP-related hemoderivatives for the treatment of mastitis [11], reproductive problems [12], and sole ulcers [13] in cows with encouraging results. The basic biology of PRP (mostly cellular and molecular studies) has been more extensively studied in horses [14–18] and to a lesser degree in dogs [19–21]. However, to the best of our knowledge, there are only two studies describing a double centrifugation tube method for concentrating bovine platelets, in which a complete cellular profile of the procured PRP was described [22] and the influence of two anticoagulants on cell concentration was also analyzed [23]. However, there is scarce information about platelet-associated growth factors such as TGF-*β*_1_ and PDGF-BB in PRPs derived from cow blood.

In the present study, we evaluated a previously described double centrifugation tube method to obtain bovine PRP [22], which consisted in spin citrated whole blood at 720*g* over 5 min. After this, the plasma obtained was divided into PRP-A (the first 50% plasma fraction nearest to the buffy coat) and PRP-B (the 50% plasma fraction over PRP-A). PRP-A was deposited in plain tubes, which were centrifugated at 720*g* over 5 min. After this, the first 25% plasma (bottom) fraction was considered as PRP-C and the remaining 75% plasma (top) fraction was considered as PRP-D.

The novelty of this research stems from the fact that we analyzed, compared, and correlated cell numbers and TGF-*β*_1_ and PDGF-BB concentrations in four different PRPs (platelet concentrates) which were produced over the entire process to obtain a final PRP fraction richer in PLTs than the other three evaluated PRP fractions or by-products. This basic study was performed to characterize from the cellular and molecular point of view four bovine PRP layers (A, B, C, and D) and to potentially recommend the use of a specific PRP for particular clinical problems in this species.

## 2. Materials and Methods

This study was approved by the committee for ethical experimentation with animals at Universidad de Caldas.

### 2.1. Animals

Nineteen clinically healthy lactating Holstein-Friesian cows with a median age of 5.5 years (range: 2.5–12 years) were used. The animals were grazing in the highlands (2300 meters above sea level) of the central Andean region of Colombia. All the animals were fed and managed under similar technical conditions. The owner of the cows was informed on the nature of the study and signed an authorization accordingly.

### 2.2. Blood Collection

Blood was collected from each cow through a 2′′ 14 G i.v. catheter (NIPRO14GX2′′, Nipro, São Paulo, Brazil), which was fixed with sutures in one of the jugular veins. The catheters were closed with a latex-free plug (IN-Stopper, B. Braun, Melsungen, Germany) and washed with 5 mL heparinized serum. Blood was obtained by attaching a 21 G (Blood Collection Set, BD Vacutainer, New Jersey) butterfly catheter to the stopper [22]. The blood from each cow was deposited in 4.5 mL tubes containing 3.2% w/v sodium citrate (BD Vacutainer, New Jersey, USA). The first tube collected was discarded to avoid dilution or the biochemical effect of heparinized serum. The tubes containing blood were maintained in an icebox and transported to the laboratory where they were processed within 1 hour of being received.

### 2.3. Preparation of PRPs by Single Centrifugation Tube Method

**Figure 1:**
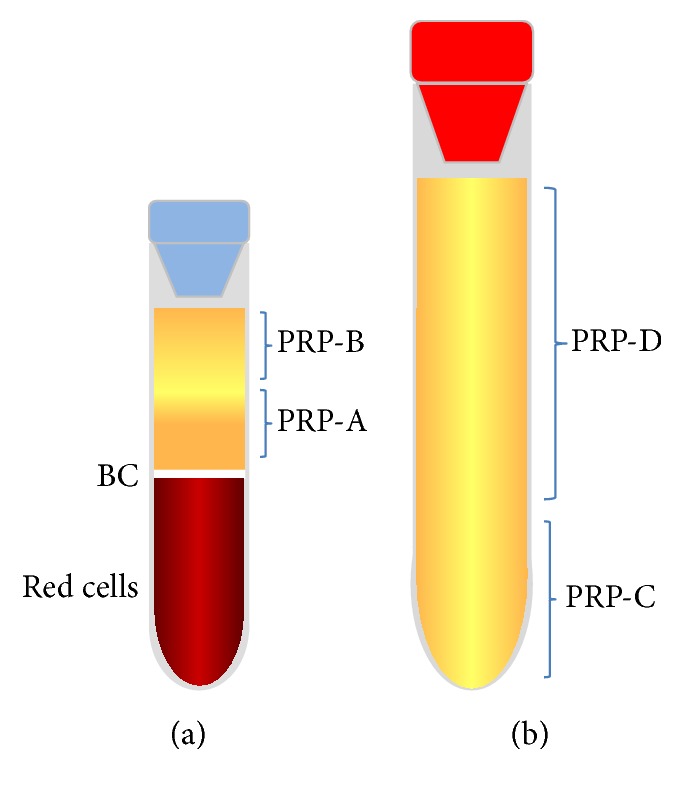
Schematic representation of the four platelet-rich plasma (PRP) fractions obtained with the single (A and B) and double (C and D) centrifugation tube methods. (a) Sodium citrate tube containing whole blood. After centrifugation, whole blood was separated in several components: red cells (PCV), buffy coat (BC), and the PRP-A (50%) and PRP-B (50%) fractions. (b) A 10 mL sterile plastic tube containing PRP-A. After centrifugation, this hemoderivative was divided into PRP-C (25%) and PRP-D (75%) fractions.

Blood tubes with sodium citrate were centrifuged (Hettich Rotofix 32, Tuttlingen, Germany) at 720*g* for 5 minutes [22]. The centrifugation process allowed for separating the blood into three phases: pack cell volume (PCV), the WBC layer, that is, the “buffy coat” (BC), and plasma. Plasma was divided into two PRP fractions: PRP-A and PRP-B. Platelet-rich plasma fraction A corresponded to the 50% of plasma closest to the BC, whereas PRP-B was considered as the 50% of plasma immediately above PRP-A (Figure 1(a)). PRP-A was collected with the needle of a 2′′ 14 G intravenous catheter (NIPRO14GX2′′, Nipro, São Paulo, Brazil), coupled to a 20 mL plastic syringe. A total of 10 mL of PRP-A and PRP-B was obtained from each cow for this part of the study.

### 2.4. Preparation of PRPs by Double Centrifugation Tube Method

20 mL of PRP-A (obtained by single centrifugation protocol) from each cow was deposited into two sterile plastic tubes without an additive (Vacuette, Greiner Bio-One, Kremsmünster, Austria), which were centrifuged at 720*g* for 5 minutes [22]. PRP-C was obtained after removing 7.5 mL of the upper plasma fraction (PRP-D) from each tube (Figure 1(b)).

### 2.5. Design of the Study

Several aliquots from PRP-A, PRP-B, PRP-C, and PRP-D were obtained for hematological and GF measurement. Samples (1 mL) from all PRPs and whole blood were employed for hematological impedance analysis (Celltac-*α* MEK 6450, Nihon Kohden, Japan), which included PLT and WBC counts and the determination of PLT-related activation parameters: mean PLT volume (MPV) and PLT distribution width (PDW). A 3 mL aliquot of each PRP was activated with 300 *μ*L of a calcium gluconate solution (9.3 mg/mL) (Ropshon Lab, Bogotá D.C., Colombia) in a 10 : 1 ratio. This procedure was done to induce PRG formation and subsequent release of GF. PRG samples were maintained with calcium gluconate under incubation at 37°C over 3 h. After this, the plasma supernatant from PRGs (clots) was put into Eppendorf tubes and stored at −80°C for later determination of GFs.

An additional 2 mL sample from all PRPs was incubated at 37°C for 15 minutes with 200 *μ*L of a solution containing 0.5% of a nonionic detergent (Triton® X-100, Sigma-Aldrich Co. LLC, MO, USA) for obtaining PRP lysates, which were used as a positive control of GF release. PRP lysates were processed in a similar fashion to PRG supernatants. Finally, 2 mL of plasma (free of cells) from each cow was also obtained by centrifugation of one blood tube at 3500*g* for 5 min. This substance was considered as the negative control for GF enrichment. Plasma samples were stored in the same fashion as PRG supernatants and PRP lysates.

### 2.6. Determination of TGF-*β*_1_ and PDGF-BB Concentrations by ELISA

The PDGF-BB and TGF-*β*_1_ concentrations in PRG supernatants, PRP lysates, and plasma were determined in duplicate by sandwich ELISA developed with commercial antibodies for human TGF-*β*_1_ (Human TGF-*β*1, DY240E, R&D Systems, Inc., MN, USA) and PDGF-BB (Human PDGF-BB, DY220, R&D Systems, Inc., MN, USA), because these mammalian GFs are highly homologous between both species (90% identity or more) and similar ELISA antibodies have been used to measure these polypeptides in cow samples [24–27]. Both ELISAs were performed according to the manufacturer's instructions. Readings were performed at 450 nm. Results are presented as pg of GF per mL of hemoderivative evaluated.

### 2.7. Statistical Analysis

Data were analyzed using statistical software (SPSS 18.0, IBM, Chicago, IL, USA). The Shapiro-Wilk test (SW) was used to evaluate the normality of the data. PLT and WBC counts and MPV and PDW parameters in whole blood and PRPs showed a normal distribution (SW, *p* > 0.05) and were compared by one-way ANOVA, followed when necessary by a Tukey test. PDGF-BB concentrations in all hemoderivatives exhibited a parametric distribution (SW, *p* > 0.05). TGF-*β*_1_ concentrations in some hemoderivatives presented a nonparametric distribution (SW, *p* < 0.05). These data were analyzed after a log⁡(*Y*) transformation. GF concentrations were compared by a generalized linear model (GLM) because there were some missing data for the concentration of both GFs. Notably, this procedure groups the dependent variables by their pattern of missing values across observations so that sums and cross-products can be collected in the most efficient manner. A Tukey test was used as a post hoc test when necessary. Furthermore, all variables were analyzed for general and specific correlations using a Pearson (*r*_*s*_) test. A *p* value ≤ 0.05 was considered significant for all the tests.

## 3. Results

### 3.1. Hematological Findings in Whole Blood and PRPs

**Figure 2:**
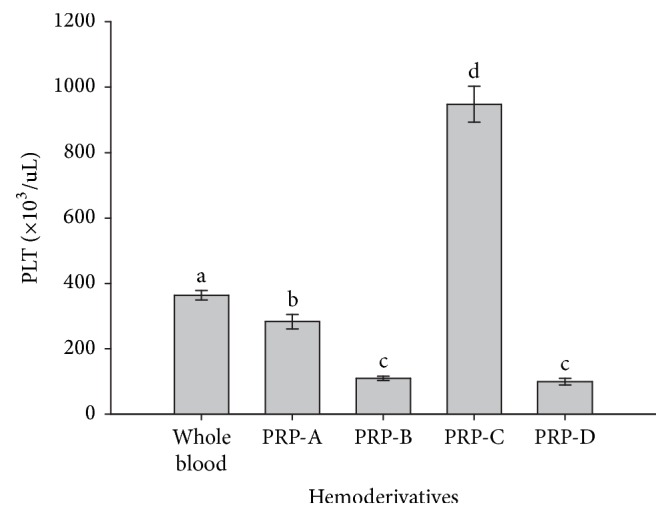
Mean (±standard deviations [SD]) of platelet (PLT) concentration (×10^3^/*μ*L) in whole blood and four PRP fractions (A, B, C, and D). a–d: different lowercase letters denote significant differences by the Tukey test (*p* = 0.001).

**Figure 3:**
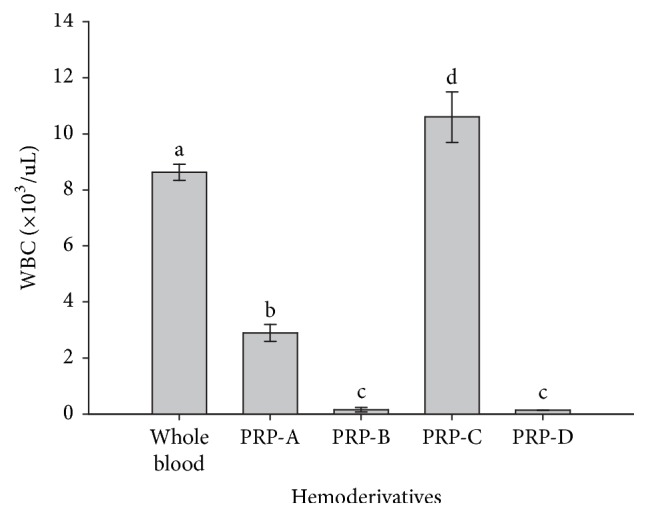
Mean (±SD) of white blood cell (WBC) concentration (×10^3^/*μ*L) in whole blood and four bovine PRP fractions (A, B, C, and D). a–d: different lowercase letters denote significant differences by the Tukey test (*p* = 0.001).

PLT counts were significantly (*p* = 0.001) different between whole blood and all PRPs. PRP-C showed a significantly (*p* = 0.001) higher PLT concentration when compared to whole blood and the other PRPs. PRP-A showed a significantly (*p* = 0.001) lower PLT concentration when compared to whole blood and PRP-C and a higher concentration in comparison to PRP-B and PRP-D, in which PLT counts were similar (Figure 2). On the other hand, WBCs showed a similar concentration pattern in whole blood and PRPs, which was very similar to those results' pattern observed for PLTs counts for the same hemoderivatives (Figure 3).

**Figure 4:**
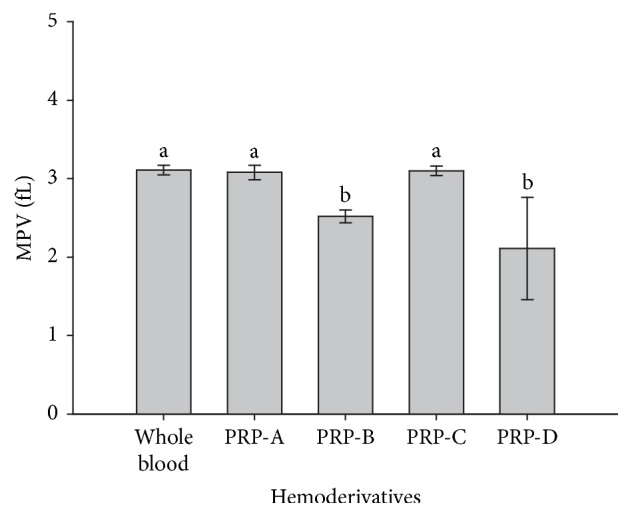
Mean (±SD) of mean platelet volume (MPV) (fL) in whole blood and four bovine PRPs (A, B, C, and D). a-b: different lowercase letters denote significant differences by the Tukey test (*p* = 0.001).

**Figure 5:**
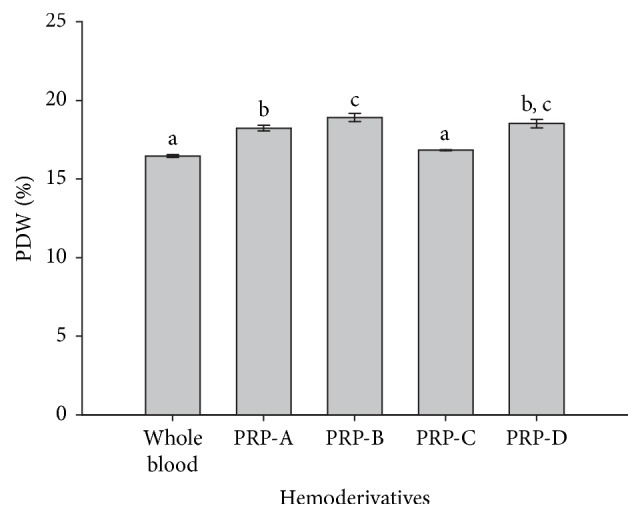
Mean (±SD) of platelet distribution width (PDW) (%) in whole blood and four bovine PRPs (A, B, C, and D). a–c: different lowercase letters denote significant differences by the Tukey test (*p* = 0.001).

MPV values were not different between whole blood, PRP-A, and PRP-C. However, the values for the same parameter were significantly (*p* = 0.001) lower for PRP-B and PRP-D when compared to the other hemoderivatives and whole blood (Figure 4). On the other hand, PDW values were significantly (*p* = 0.001) higher in PRP-B and PRP-D in comparison to whole blood and PRP-C, whereas PDW values were not different between PRP-B and PRP-D and between whole blood and PRP-C (Figure 5).

### 3.2. TGF-*β*_1_ and PDGF-BB Concentrations in Plasma, PRG Supernatants, and PRP Lysates

**Figure 6:**
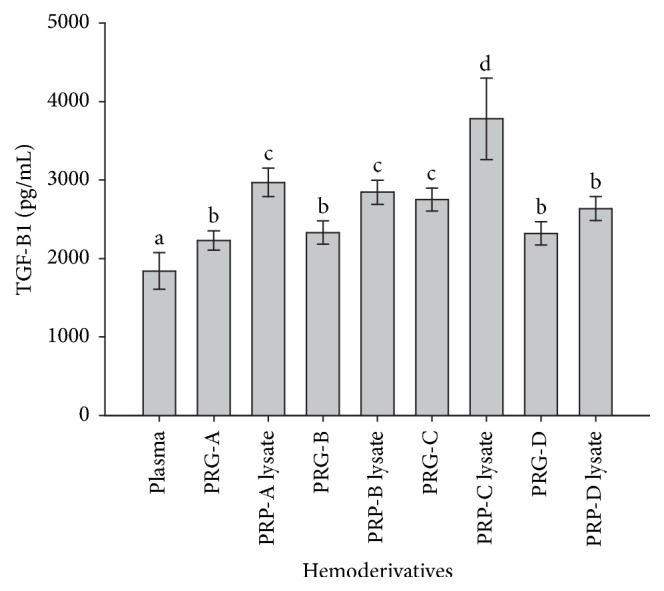
Mean (±SD) of TGF-*β*_1_ concentration (pg/mL) in plasma and four bovine platelet-rich gel (PRG) supernatants (A, B, C, and D) and their respective PRP lysates (A, B, C, and D). a–d: different lowercase letters denote significant differences by the Tukey test (*p* < 0.05).

TGF-*β*_1_ concentrations were significantly (*p* = 0.001) lower in plasma when compared with the other hemoderivatives. On the other hand, the PRP-C lysate showed the highest TGF-*β*_1_ concentration when compared to the rest of the hemoderivatives. Supernatants from PRG-A, PRG-B, and PRG-D were not different for this GF concentration. When each PRG supernatant was compared for its TGF-*β*_1_ concentration in relation to the respective PRP lysate, we found significant differences between the PRG-A supernatant and PRP-A lysate (*p* = 0.003), the PRG-B supernatant and PRP-B lysate (*p* = 0.001), and the PRG-C supernatant and PRP-C lysate (*p* = 0.0001). Notably, the TGF-*β*_1_ concentration in the PRG-C supernatants was significantly (*p* = 0.0001) higher in comparison with the other PRG supernatants (Figure 6). Missing values for this GF were observed for 2% of the generated data.

**Figure 7:**
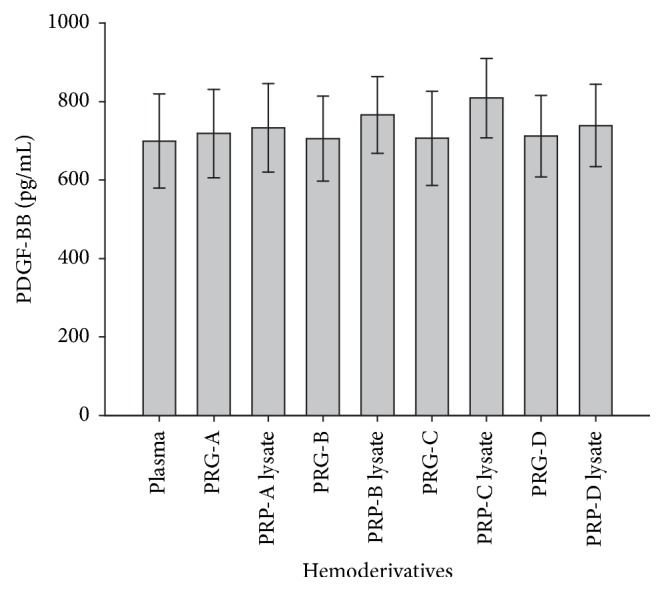
Mean (±SD) of PDGF-BB concentration (pg/mL) in plasma and four bovine PRG supernatants (A, B, C, and D) and their respective PRP lysates (A, B, C, and D).

PDGF-BB concentrations remained at a concentration oscillating between 600 and 850 pg/mL in all hemoderivatives evaluated. No significant differences were noted for the concentration of this GF between the hemoderivatives (Figure 7). Missing values for this GF were observed for 3% of the generated data.

### 3.3. Correlations

Significant correlations (*r*_*s*_) were noted between PLT counts and WBC counts (*r*_*s*_ = 0.80, *p* = 0.0001), as well as between PLT counts and PDW (*r*_*s*_ = −0.60, *p* = 0.001). Several significant weak correlations were noted between WBC counts and PDGF-BB concentrations (*r*_*s*_ = −0.50, *p* = 0.001), between PLT counts and PDGF-BB concentrations (*r*_*s*_ = −0.40, *p* = 0.001), and between PDGF-BB and TGF-*β*_1_ concentrations (*r*_*s*_ = 0.50, *p* = 0.001).

## 4. Discussion

The study described here presents novel and complimentary [22, 23] information on the double centrifugation tube technique for producing bovine PRP. As mentioned above, to the best of our knowledge, there are two additional published techniques [11–13] for concentrating platelets in cattle. However, these studies did not provide information about WBC and GF concentrations. This information is of paramount importance to establish PRP protocols and, consequently, to understand their potential biological effects on cells and tissues [2, 28–30].

Currently, PRP classification is an important point of discussion for clinicians and researchers, because the cellular composition of a particular PRP will determine the content of GF and cytokines and consequently its biological effect [2, 28, 30]. Notably, 16 years ago, when seminal research work on PRP was published, there was evident interest in considering PRP as a 5 mL solution containing at least 1000 × 10^3^ PLTs/*μ*L [31]. At that time, it was thought that higher PLT concentrations in PRP would produce better clinical results than a substance with a lower PLT concentration. However, this paradigm has changed over time and, currently, it is widely accepted that PRP with a low PLT concentration (≥1.2 times greater than the PLT concentration in whole blood) can also produce good clinical results when compared to PRPs very rich in PLTs [32].

The results from the present study, particularly regarding the WBC concentration in PRPs, allowed us to classify the hemoderivatives into three types of liquid PRP compounds: (1) Lc-PRP (PRP-C), (2) Lr-PRP (PRP-B and PRP-D) [2], and (3) transitional PRP (T-PRP) (PRP-A). This last type of PRP is proposed as a PRP product characterized by a low count of WBCs (0.3–0.5 times the WBC concentration in whole blood) and a PLT concentration similar or slightly lower or higher in relation to the PLT concentration in whole blood [33]. At this point, it remains necessary to evaluate these bovine PRPs in* in vitro* systems and under clinical conditions affecting cattle. In general, these substances could be used as a treatment for orthopedic lesions, solar ulcers, mastitis, and traumatic wounds or as a surgical coadjuvant in this species.

PRP-C produced with the method evaluated in the present study presented a slightly lower concentration of PLTs (947 × 10^3^ PLTs/*μ*L) when compared with the method described by Lange-Consiglio et al. [11, 12] who achieved 1000 × 10^3^ PLTs/*μ*L. Furthermore, this same hemoderivative presented a lower concentration of PLTs when compared with the technique developed by Tsuzuki et al. [13] who achieved 1528 × 10^3^ PLTs/*μ*L. On the other hand, the other PRP layers (A, B, and D) evaluated in the present study presented lower PLT concentrations in comparison with those obtained in the aforementioned studies [11–13] and even in comparison to the PLT counts in whole blood of the same cows. Unfortunately, the WBC concentration was not reported for the techniques used for producing PRPs in cattle [11, 12, 29], which prevents us from comparing our findings with those reports.

MPV and PDW are frequently used parameters for indicating PLT activation in humans [34, 35], horses [17, 36], dogs [37, 38], and cattle [39], among others. These parameters are useful to determine PRP quality. However, the values for these parameters could change as a function of the anticoagulant used for blood collection and the technology of the device used for measurement [15, 38, 40, 41]. Notably, MPV (fL) values in whole blood (3.11 ± 0.06) from the cows used in this study were lower than the same parameter reported in healthy cows in which whole blood (6.30 ± 0.17) was obtained with EDTA [39]. However, PDW (%) values in whole blood (16.47 ± 0.10) from our cows were higher than the same value reported for whole blood (10.70 ± 0.30) of healthy cows using EDTA [39].

MPV values for all PRP layers evaluated in this study were similar between whole blood and PRP-A and PRP-C and significantly lower in PRP-B and PRP-D. This situation has also been observed in PRPs from horses using a similar technique [3]. One explanation for the changing MPV values, especially those related to PRP-B and PRP-D, is that these hemoderivatives presented a lower concentration of WBCs and erythrocytes. It is well known that PLTs become activated (reversibly) when they are centrifuged in the presence of these cells [42].

On the other hand, PDW (%) values were significantly lower in whole blood and PRP-C in comparison with the other hemoderivatives. We also found a negative correlation (−0.6) between this parameter and PLT counts in the PRPs. Possibly, PDW could be a more sensible indicator of PLT activation (PRP quality) than MPV in cattle, because an increase in this parameter was related to a lower concentration of PLTs in PRPs from the cows used in this study. It is important to consider that in humans PDW is a more specific marker for platelet activation than MPV, because it does not increase with only platelet swelling [41].

The inclusion of a negative control (plasma) and a positive control (PRP lysate) for establishing GF enrichment allowed us to observe that the supernatants from PRG-C and their correspondent PRP lysate showed the highest TGF-*β*_1_ concentrations. Notably, 73% of TGF-*β*_1_ stored in PLTs and WBCs from PRP-C was massively released 3 h after activation. It seems that TGF-*β*_1_ release from cattle PRP after its activation is more massive and rapid than in horses [17]. Notably, we found that TGF-*β*_1_ concentrations were significantly higher in all hemoderivatives when compared to plasma. This finding suggests that all PRP layers evaluated contain important TGF-*β*_1_ concentrations, indicating that these biomaterials have potential clinical application in this species.

PDGF-BB concentrations were similar between all hemoderivatives evaluated in the present study. This was an unexpected biological phenomenon, because this GF is regularly present in lower concentrations in plasma and in higher concentrations in PRG supernatants and their correspondent PRP lysates in mammals, like horses [15, 17, 43], cats [44], and rabbits [33]. Two technical or biological explanations could explain this finding: (1) human PDGF-BB specific antibodies were used for capturing bovine PDGF-BB. As mentioned above, this GF is very similar between human and bovine [25]; however, similar PDGF-BB ELISA antibodies (from the same manufacturer) have been used for the same objective in other studies in which bovine PDGF-BB concentrations were measured [26, 27]. (2) It is possible that bovine PDGF-BB presented a high plasma concentration and a nonvariable concentration in PRP as a function of the PLT concentration. Notably, this biological phenomenon has also been observed in humans [45, 46] and horses [43, 47] for an important anabolic GF related to PRP, insulin growth factor type I. However, further research evaluating the concentrations of several bovine PDGF isoforms in PRP using species-specific ELISA antibodies should be performed.

## 5. Conclusions

This study demonstrates that several bovine PRPs can be obtained using single or double centrifugation tube methods. According to the results on cell counts, the PRP types could be classified as Lc-PRP (PRP-C), Lr-PRP (PRP-B and PRP-D), or transitional PRP (T-PRP) (PRP-A). PRP-C presented the highest concentration of PLTs, WBCs, and TGF-*β*_1_, followed by PRP-A, PRP-B, and PRP-D. However, PDGF-BB concentrations were similar between all hemoderivatives.
